# ERBB Receptors and Their Ligands in the Developing Mammary Glands of Different Species: Fifteen Characters in Search of an Author

**DOI:** 10.1007/s10911-023-09538-w

**Published:** 2023-05-23

**Authors:** Alessia Morato, Paolo Accornero, Russell C. Hovey

**Affiliations:** 1 Department of Animal Science, University of California, Davis, One Shields Avenue, Davis, CA 95616 USA; 2 Department of Veterinary Science, University of Turin, Largo Paolo Braccini 2, Grugliasco, TO 10095 Italy

**Keywords:** Hormone, Epidermal growth factor, Stroma, Amphiregulin

## Abstract

The ERBB tyrosine kinase receptors and their ligands belong to a complex family that has diverse biological effects and expression profiles in the developing mammary glands, where its members play an essential role in translating hormone signals into local effects. While our understanding of these processes stems mostly from mouse models, there is the potential for differences in how this family functions in the mammary glands of other species, particularly in light of their unique histomorphological features. Herein we review the postnatal distribution and function of ERBB receptors and their ligands in the mammary glands of rodents and humans, as well as for livestock and companion animals. Our analysis highlights the diverse biology for this family and its members across species, the regulation of their expression, and how their roles and functions might be modulated by varying stromal composition and hormone interactions. Given that ERBB receptors and their ligands have the potential to influence processes ranging from normal mammary development to diseased states such as cancer and/or mastitis, both in human and veterinary medicine, a more complete understanding of their biological functions should help to direct future research and the identification of new therapeutic targets.

## Introduction

In his most acclaimed play *Six Characters in Search of an* *Author,* the Italian dramatist Luigi Pirandello brings six people to a stage where a company is rehearsing. These characters, who belong to the same extended family, claim to be “incomplete” and demand the director of the play to listen to their stories. An intricate family drama arises, ultimately leaving the director confused and the truth uncertain. In this review the characters are fifteen and the stage is the mammary gland. As for the mentioned author, the combined efforts of many scientists is needed to unravel the truth….

**Fig. 1:**
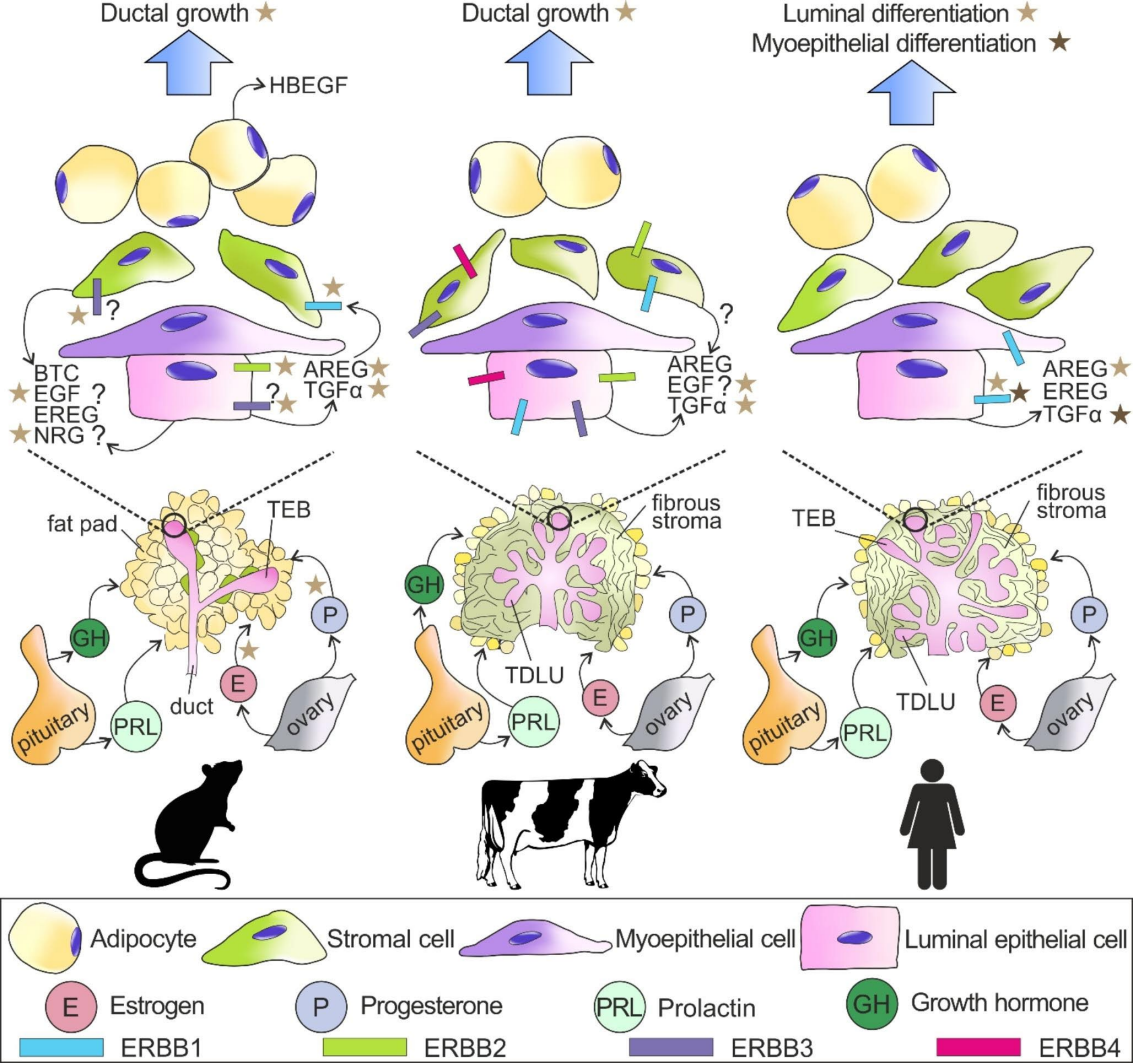
Mammary gland histomorphology and the epidermal growth factor (EGF) receptor (ERBB) family of receptors and their ligands in mice (left), ruminants (middle) and humans (right) during puberty and sexual maturity. The main systemic hormones involved during pre-gestational development are summarized. Asterisks link individual ERBB receptors to specific biological effects (blue arrows). Question marks represent incomplete information regarding ERBBs and their ligands. Abbreviations = AREG, amphiregulin; BTC, betacellulin; EGF, epidermal growth factor; EREG, epiregulin; HBEGF, heparin-binding EGF; NRG, neuregulin; Pituitary, pituitary gland; TEB, terminal end bud; TDLU, terminal ductal lobular unit; TGFα, transforming growth factor-α. Abbreviations for graphics are described in the figure. Figure created with CorelDraw X8.

The mammary glands are unique in that they undergo a large proportion of their development after puberty under the influence of the reproductive hormones, where estrogen (E) directs ductal elongation into the surrounding stroma, while progesterone (P) and prolactin (PRL) promote branching, alveologenesis and lactogenesis. Despite the essential role of steroid hormones during mammary morphogenesis, only a subpopulation of mammary epithelial cells (MEC) are hormone receptor positive (HR+) due to their expression of receptors for E and P. A conclusion from genetically modified mice is that HR- epithelial cells, stromal cells or adipocytes rely on HR+ MEC to translate endocrine signals into paracrine cues [1, 2]. The mammary parenchyma in mice is very simple in its structure and originates from a branched epithelial tree surrounded by a predominantly adipose stroma (Fig. 1). Club shaped structures referred to as terminal end buds (TEBs) coordinate elongation of the mammary epithelium in pubescent mice. Similar structures are absent in cows, sheep and goats (collectively referred to as ruminants), where their mammary glands are characterized by complex ductal-lobular epithelial structures referred to as terminal ductal lobular units (TDLU), a more fibrous stroma, and less proximity to adipocytes (reviewed by Hovey et al. [3]). The mammary glands of pigs and humans have the combination of murine and ruminant elements, namely both TEB and TDLU, surrounded by a primarily fibrous stroma [4, 5]. The co-presence of TEB and TDLU has also been described in the mammary glands of dogs [6]. Several lines of evidence suggest that hormonal stimuli acting via local regulators underlie this strikingly-diverse morphological variation [7].

Central to this local growth regulatory mechanism is the family of ERBB receptors and their ligands [8]. The ERBB family comprises four known receptors and numerous binding factors that are dynamically expressed throughout mammary gland development in mice [9] and are essential for postnatal mammary development. For instance, amphiregulin (AREG) mediates the actions of E at the onset of puberty by activating EGFR in the mammary stroma [10]. In a similar way, activation of ERBB4 is indispensable during lobuloalveolar development and lactogenesis [11]. Nevertheless, the role of ERBB receptors and their ligands has not been investigated for all stages of development, where numerous gaps remain, including for their spatial distribution. Likewise, the literature has an abundance of data for rodents relative to other species that have important and unique histomorphological characteristics [12].

Here we provide a comparative review of the expression and function of the ERBB receptors and their ligands during various stages of postnatal mammary development across several species. We also review how interactions between systemic hormones, ERBB receptors and various signaling pathways can impact development of the mammary gland and may account for differences in its morphology across species. By highlighting the paucity of data available for non-rodent species, we seek to convey that a thorough appreciation of ERBB biology in the mammary glands of different animal models is a necessary and overdue background for future research in both mammary physiology and pathology.

## The Protagonists: The ERBB Receptors

Many excellent reviews detail the structure and activation of ERBB receptors elsewhere [13–15]. That said, there are several key points about the ERBB receptors with regard to their signaling versus other receptor tyrosine kinases that are worth highlighting. First, the EGF/ERBB family is notably large, having 11 known EGF-like growth factors that can variably bind 4 ERBB receptors. Second, the ERBBs are exclusively activated by either homo- or heterodimerization. Finally, a wide range of signaling pathways can be triggered in response to different ERBB homo- and heterodimers, leading to diverse outcomes. Aspects of these features are described below, with a focus on their implications for mammary biology.

### Structure of the ERBB Receptors

**Fig. 2:**
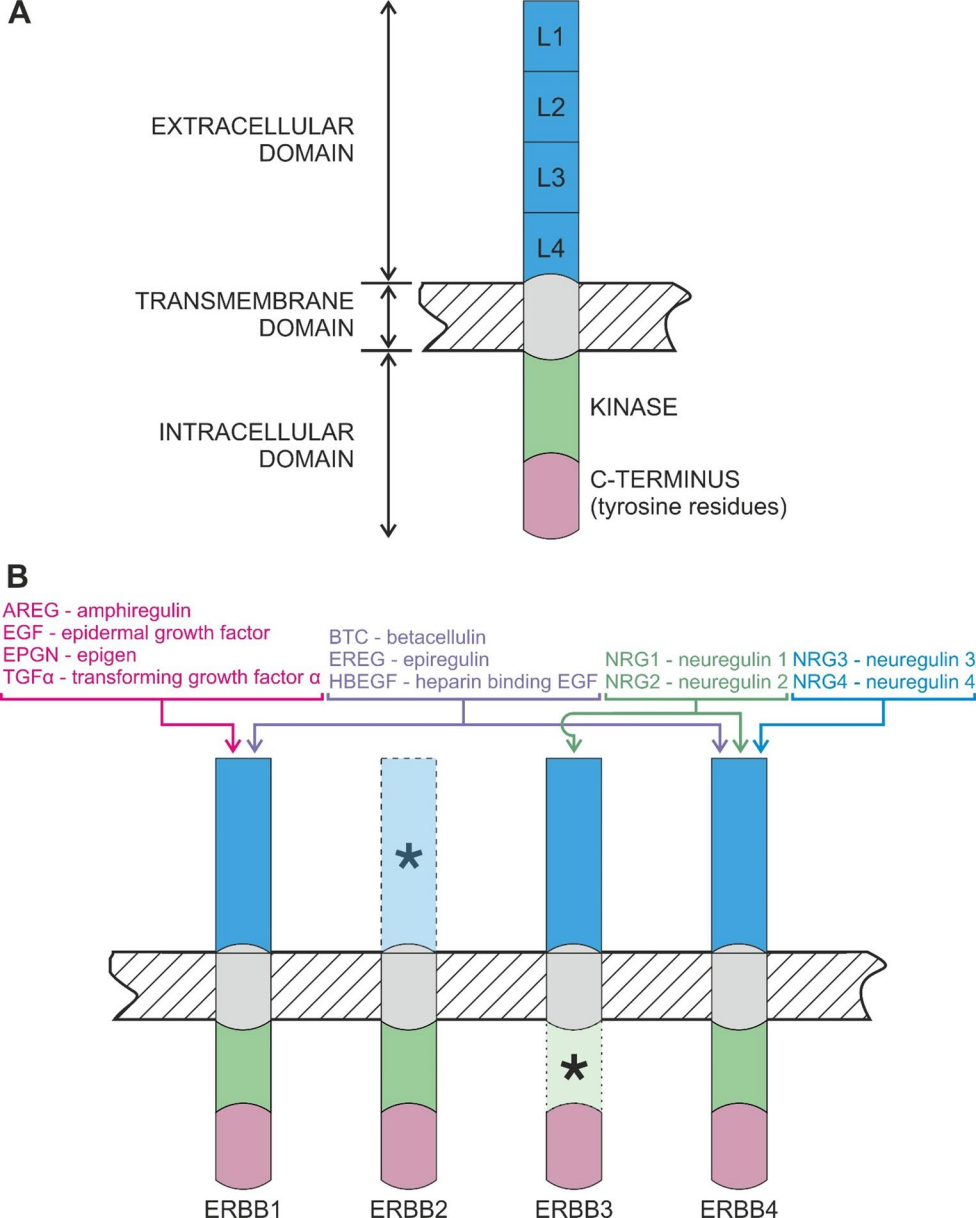
(**A**) Structure of the epidermal growth factor (EGF) receptor (ERBB1), the archetype of the ERBB family. (**B**) Binding potential of EGF-like factors. Blue, extracellular region divided into L1-4 domains; green, intracellular tyrosine kinase domain; purple, intracellular C-terminus containing phosphorylatable tyrosine residues. Asterisks = defective extracellular domain of ERBB2, and defective intracellular domain of ERBB3. Figure created with CorelDraw X8.

The ERBB1/EGFR is an archetypal transmembrane protein that can activate tyrosine kinase activity. ERBB1 is a 170 kDa protein with a glycosylated extracellular domain that contains 4 subdomains arranged in tandem [16, 17], namely the homologous subdomains I and III (L1 and L2), and the cysteine-rich homologous subdomains II and IV (CR1 and CR2) [18–20]. Subdomain III is primarily involved in ligand binding (Fig. 2A). The other regions of ERBB1 include a single transmembrane domain, and an intracellular tyrosine kinase domain that confers the ability to activate autophosphorylation. Importantly, ERBB1 can translocate to the nucleus and function as a transcriptional regulator, or can interact with transcriptional factors such as signal transducer and activator of transcription (STAT) 3 and STAT5 [21], although the role of this mechanism during mammary morphogenesis has not been clarified. The ERBB2/human epidermal growth factor receptor 2 (HER2)/neu (185 kDa) is commonly referred to as orphan receptor: while it has an intrinsic kinase activity, the extracellular domain of ERBB2 has a permanent active conformation that prevents ligand binding (Fig. 2B), while also ensuring that the dimerization loop of subdomain IV remains exposed. The third family member, ERBB3 (160/180 kDa), has a defective cytoplasmic domain due to the absence of several key residues (Fig. 2B), which gives this receptor only 1/1000th the kinase activity of ERBB1 [22]. As a result, a role for either ERBB2 or ERBB3 at any stage of mammary development always implies their heterodimerization with another partner. Conversely, ERBB4/HER4 (180 kDa) can form both homodimers and heterodimers with other ERBB receptors, and can consequently undergo proteolytic processing to generate an 80 kDa cleaved intracellular region, which can translocate to the nucleus to regulate gene expression [23]. As will be discussed later, both the entire ERBB4 and the cleaved intracellular region have two isoforms (Cyt-1 and Cyt-2 for the cleaved domain; or JM-a, Cyt-1, and Cyt-2 for the full-length receptor) that confer the ability of ERBB4 to mediate both proliferative and differentiative processes in the developing gland.

### ERBB Dimerization and Activation

Signaling downstream of the various ERBBs is triggered by ligand binding and subsequent receptor homo/heterodimerization [24, 25]. Only ERBB1 and ERBB4 have the ability to homodimerize to initiate signaling [26], whereas ERBB2 and ERBB3 can only heterodimerize [27–29]. As a result, ten different ERBB dimers are possible. Within these combinations, ERBB2 is the preferred partner for all other ERBB receptors. For example, ERBB2 and ERBB1 dimerize with the same affinity as the ERBB1 homodimer [30], and overexpression of ERBB2 and its heterodimerization with ERBB3 are both essential to induce breast tumor cell proliferation [31].

Due to the rigidity of its extracellular region, ERBB1 can only exist in an inactive (tethered) or active (extended) conformation. In the inactive form, subdomains II and IV are in close contact, whereas subdomains I and III are spaced apart, forming a potential ligand-binding “pocket”. After ligand binding, the ERBB1 “clicks” to its active form wherein subdomains I and III align, whereas subdomain II moves away from subdomain IV to expose its dimerization loop. Binding between the loops on the two dimerizing receptors induces the phosphorylation of intracellular residues. Each ERBB has a characteristic collection of phosphorylatable tyrosine residues (reviewed by Roskoski [32]). In addition, ERBB ligands have different affinities for ERBB receptors and trigger the phosphorylation of different sets of tyrosine residues within the intracellular non-catalytic region [33].

### The ERBB Ligands

Eleven EGF-like peptide growth factors share a range of structural and functional properties. This family can be divided into three groups based on their binding affinity for one or more of the ERBB receptors [34]. One group of ligands, including epidermal growth factor (EGF), transforming growth factor alpha (TGFα), AREG and epigen (EPGN), can only bind ERBB1. The second group binds both ERBB1 and ERBB4, and includes epiregulin (EREG), betacellulin (BTC) and heparin-binding EGF (HBEGF), while the third group includes the neuregulins (NRG) that play a crucial role in neural development. Among these ligands, NRG1 and NRG2 can bind ERBB3 and ERBB4, whereas NRG3 and NRG4 can only bind ERBB4 (Fig. 2B).

The EGF-like ligands are expressed as type I transmembrane precursor proteins comprising an extracellular component, a transmembrane segment and a short intracellular region [35, 36]. The extracellular region includes one or more EGF-like motif(s), consisting of a hexameric cysteine sequence that creates three intramolecular disulfide bonds. This motif is essential and sufficient for ERBB-binding [37, 38]. In addition, the extracellular region of AREG and HBEGF includes an amino-terminal heparin-binding domain. The extracellular domain of ERBB ligands can be cleaved by various members of the A disintegrin and metalloproteinases (ADAMs) in a process referred to as protein ectodomain shedding [39, 40]. While the proteolytic release of this domain is generally necessary for binding ERBB receptors, some ligands that share structural similarities with HBEGF are biologically active even when bound to the membrane [41].

## Distribution of ERBB Receptors and Ligands on the Mammary Stage

### Mice

#### Puberty

Edery et al. [42] first reported the expression of two classes of EGF-binding receptors that had either low or high EGF-binding affinity in the epithelial and stromal compartment of the mammary glands in nulliparous mice, respectively. Receptor levels were highest in the glands of nulliparous mice, except for another peak during mid-gestation. A detailed profile of receptor expression then became available once the *Erbb* genes were identified (Table 1). The expression of *Erbb1*, *Erbb2* and *Erbb3* mRNA was often detected in the mammary glands of nulliparous females, even though only the phosphorylated form of ERBB1 and ERBB2 proteins was detected by immunoblotting of ERBB-immunoprecipitates, which raises questions about the sensitivity of these assays. The presence of *Erbb4* mRNA and the ERBB3 protein in the mammary glands of nulliparous mice remains controversial [9, 43, 44]. Based on immunohistochemical analyses, ERBB1 and ERBB2 are equally-abundant in the epithelium and stroma of the mammary glands from pubescent female mice, then respectively become predominant in the stroma and epithelium by the time of sexual maturity [9]. The expression of ERBB1 mainly localizes to stromal cells surrounding the ducts and, to a lesser extent, to the cap cells of TEBs [45].

**Table 1:** Expression and function of ERBB receptors and their ligands in the mammary glands of mice, rats and ruminants during puberty and sexual maturity. Missing receptors/ligands have either not been found or investigated (see text for details). The primary spatial and/or temporal expression patterns are indicated in brackets. Abbreviations = AREG, amphiregulin; BTC, betacellulin; EGF, epidermal growth factor; EPGN, epigen; EREG, epiregulin; HBEGF, heparin-binding EGF; MEC, mammary epithelial cells; nd, not detected; NRG, neuregulin; TGFα, transforming growth factor-α

	Species		mice		rats		ruminants		humans	
**Receptors**	**ERBB1**	**expression**	mRNA, protein (stroma)	[9, 43, 45]	Protein (TEBs)	[59]	Protein (cows, sheep)	cow [5], sheep [6]	mRNA, protein (MEC)	[107–109]
		**function**	Ductal growth in vivo	[43, 46, 124]						
	**ERBB2**	**expression**	mRNA, protein (MEC)	[9, 43]	Protein (MEC and stroma)	[58]	Protein (cows)	[68]		
		**function**	Ductal growth	[127, 128]						
	**ERBB3**	**expression**	mRNA, protein?	[9, 44]	Protein (ducts and stroma)	[58]	Protein (cows)	[68]		
		**function**	Ductal growth	[129, 130]						
	**ERBB4**	**expression**	Unclear	[9, 44]	Protein (TEBs, ducts, stroma)	[58]	Proteins (cows)	[68]		
		**function**	Inhibition of proliferation	[132, 133]						
**Ligands**	**AREG**	**expression**	Protein, mRNA (MEC)	[9, 46, 47]	mRNA (adult rats)	[64]	mRNA (sheep)	[75]	mRNA	[112]
		**function**	Ductal growth in vivo	[1]	MEC proliferation in vivo	[64]			MEC luminal differentiation	[114]
	**BTC**	**expression**	mRNA	[9]	mRNA (adult rats)	[64]				
		**function**								
	**EGF**	**expression**			Protein (MEC, stroma, fat)	[63]				
		**function**	Ductal growth *in vivo/vitro*	[45, 54, 120]			MEC mitogenesis *in vivo/vitro* (cows, sheep)	cow [140, 142–146], sheep [67, 141]	MEC myoepithelial/ luminal differentiation	[114]
	**EPGN**	**expression**	nd	[9]						
		**function**								
	**EREG**	**expression**	mRNA	[9, 47]	mRNA (adults)	[64]			mRNA	[113]
		**function**								
	**HBEGF**	**expression**	Protein, mRNA (fat cells)	[9, 48]	mRNA (adults)	[64]				
		**function**								
	**TGFα**	**expression**	Protein, mRNA (MEC)	[9, 48]	mRNA, Protein (MEC, stroma, fat)	[62, 63, 198]	Protein?	[74]	mRNA, protein	[107, 108, 112]
		**function**	ductal growth in vitro	[54, 119]			MEC mitogenesis in vitro (cows, sheep)	cow [73, 140, 142, 143], sheep [67, 141]		
	**NRG1-4**	**expression**								
		**function**	ductal growth in vivo	[46, 131]						

Using microarray analysis of the epithelium and stroma from the mammary glands of 5-week old female mice, Sternlicht and colleagues showed that of the various ERBB ligands, only *Areg* was abundant in peripubertal animals [46]. By comparison, others had previously indicated that *Btc*, *Ereg*, *Hbegf*, and *Tgfa* mRNA were expressed in the mammary glands of nulliparous female mice, and that expression for both *Areg* and *Ereg* was induced during puberty [9, 47]. While mRNA transcripts and protein for *Areg* and *Tgfa* predominate in the epithelium, HBEGF has been detected in both the epithelium and adipocytes [48].

#### Gestation

During gestation, the mammary glands of mice undergo a massive increase in secondary and tertiary ductal branching followed by the progression of alveolar buds into mature alveoli. Transcripts for all ERBB receptors have been identified in the glands of pregnant mice [9, 44, 49, 50] (Table 2). Compared to nulliparous females, approximately the same level of *Erbb1*, *Erbb2* and *Erbb3* gene expression was sustained in the mammary glands during gestation, while *Erbb4* gene expression was induced at the onset of gestation [51, 52]. As gestation progresses, the abundance of all ERBB proteins increases in the mammary glands, where ERBBs are primarily expressed in the epithelium, and are either scarce or absent in the stroma [9, 44, 49].

**Table 2:** Expression and function of ERBB receptors and ligands in the mammary glands of mice, rats and ruminants during gestation. Missing receptors/ligands have either not been found or investigated (see text for details). The primary spatial and/or temporal expression patterns are indicated in brackets. Abbreviations = AREG, amphiregulin; BTC, betacellulin; EGF, epidermal growth factor; EREG, epiregulin; HBEGF, heparin-binding EGF; MEC, mammary epithelial cells; NRG, neuregulin; TGFα, transforming growth factor-α. Lobuloalveolar (LA) growth = increased number of LA and/or proliferation of the alveolar epithelium. LA distension = cavitation of LA. LA differentiation = LA displaying histological signs of secretory activity. Milk protein expression = gene or protein expression

	Species		mice		rats		ruminants		humans	
**Receptors**	**ERBB1**	**expression**	mRNA, protein (ducts)	[9]	Protein	[59]	Protein (cow, sheep)	[66, 67]		
		**function**								
	**ERBB2**	**expression**	mRNA, protein (ducts)	[9, 51]	Protein (ducts, stroma)	[58]				
		**function**	LA distention	[51]						
	**ERBB3**	**expression**	mRNA, protein (luminal MEC)	[9, 44]	Protein (ducts, stroma)	[58]				
		**function**	LA growth	[49, 129]						
	**ERBB4**	**expression**	mRNA, protein (ducts)	[9, 44]	Absent	[58]				
		**function**	LA growth, differentiation	[50, 153, 154]						
**Ligands**	**AREG**	**expression**	mRNA	[9]			mRNA, protein (sheep)	[75]		
		**function**	In vivo LA differentiation	[48]			In vitro MEC mitogenesis (sheep)	[141]		
	**BTC**	**expression**	mRNA	[9]						
		**function**								
	**EGF**	**expression**	mRNA	[9]			Protein (goat mammary secretion)	[70]		
		**function**	In vitro MEC mitogenesis, milk protein expression. In vivo LA growth	[48, 121, 147, 152, 199, 200]			Mammary growth. *In vitro-vivo* DNA synthesis	[143, 160]		
	**EREG**	**expression**	mRNA	[9]						
		**function**								
	**HBEGF**	**expression**	mRNA	[9]						
		**function**								
	**TGFα**	**expression**	mRNA (decreasing)	[9, 47]	mRNA, protein (MEC, stroma)	[62, 198]	Protein (cow)	[72, 73]	mRNA	[62]
		**function**	LA growth	[48, 200]			Mammary growth. *In vitro-vivo* DNA synthesis	[143, 160] (cow) [141] (sheep)		
	**NRG1-4**	**expression**	mRNA	[9]						
		**function**	*In vitro-vivo* LA growth-distention. In vitro milk protein expression.	[44, 156]						

Expression of mRNA for the ligands *Areg*, *Btc*, *Ereg*, *Hbegf* and *Tgfa* is abundant in the mammary glands of mice at the onset of gestation. While *Areg* mRNA abundance decreases thereafter, *Btc*, *Ereg* and *Tgfa* expression are only downregulated at the end of gestation, while *Hbegf* and *Nrg1* levels are maintained until parturition. More specifically, *Nrg1* gene expression peaks at day 15 of gestation in mice, while the level of TGFα protein decreases, which was hypothesized to stimulate the onset of whey acidic protein (WAP) expression [47, 53]. Compared to other ligands, the expression of *Egf* mRNA within the mammary glands progressively increases from mid-gestation [9].

#### Lactation and Involution

Abundance of transcripts encoding all four ERBB receptors generally decreases in the mammary gland during lactation (Table 3). Among the ERBB ligands, only the expression of *Egf* mRNA was consistently elevated within the mammary glands throughout lactation [9, 54, 55], where an EGF precursor was mostly detected within lactating alveolar cells in the apical part of the membrane [56]. During involution, there is a general re-expression of all ERBB receptors, and of *Tgfa*, *Hbegf*, *Btc* and *Nrg1* mRNA [9].

**Table 3:** Expression and function of ERBB receptors and ligands in the mammary glands of mice, rats and ruminants during lactation. Missing receptors/ligands have not been either found or investigated (see text for details). Data for humans is not included due to a lack of information. The primary spatial and/or temporal expression patterns are indicated in brackets. Abbreviations = AREG, amphiregulin; EGF, epidermal growth factor; MEC, mammary epithelial cells; NRG, neuregulin; TGFα, transforming growth factor-α. Lobuloalveolar (LA) growth = increased number of LA and/or alveolar epithelial proliferation. LA distension = cavitation of LA. LA differentiation = LA displaying histological signs of secretory activity. Milk protein expression = gene or protein expression

	Species		mouse		rat		ruminants	
**Receptors**	**ERBB1**	**expression**	mRNA (decreasing), protein	[9]	Protein (decreasing)	[59]	Protein (cows)	[66]
		**function**	LA differentiation	[163]				
	**ERBB2**	**expression**	mRNA (decreasing), protein	[9]	Protein?	[58, 60, 61]		
		**function**	Milk protein expression, LA distention	[51]				
	**ERBB3**	**expression**	mRNA (decreasing), protein	[9]	Almost absent	[58]		
		**function**	Milk protein expression, LA distention	[130]				
	**ERBB4**	**expression**	mRNA (decreasing), protein	[9]	Almost absent	[58]		
		**function**	Milk protein expression, LA distention	[50, 52, 153]				
**Ligands**	**AREG**	**expression**					mRNA, Protein (sheep)	[75]
		**function**						
	**EGF**	**expression**	mRNA, protein	[9, 54–56]				
		**function**					In vitro MEC mitogenesis (sheep)	[141]
	**TGFα**	**expression**			mRNA, protein (MEC, stroma?)	[62, 198, 201]		
		**function**					In vitro MEC mitogenesis (sheep)	[141]
	**NRG1-4**	**expression**						
		**function**	In vivo LA growth-distension, milk protein expression	[156]				

### Rats

The distribution of ERBB receptors and ligands in the mammary glands of rats warrants a separate description given that development of their glands differs subtly to that in mice (Table 1–3). Several studies have reported the differential expression of ERBB receptors in rats across the course of mammary development [57–60]. According to Darcy et al. [58, 59], proteins for all ERBB receptors are expressed in the mammary glands of peripubertal rats. Of note, ERBB1 was primarily expressed in TEBs, which contrasts to findings in mice, while ERBB3 was primarily expressed in the ducts and stroma. During puberty, ERBB4 was mostly expressed in TEBs, the surrounding stroma, and ducts. By contrast, ERBB2 was evenly distributed across epithelial and stromal compartments, consistent with its putative role as the preferred heterodimerization partner for other ERBB receptors. Interestingly, Dati et al. did not detect ERBB2 in the mammary glands of nulliparous rats, perhaps because its levels were below the detection limit of the immunoblot analysis. All the six possible heterodimers of ERBB1-4 were detected in the mammary glands of adult nulliparous rats [58].

During gestation, ERBB1, -2 and − 3 could be found in both the mammary epithelium of the ducts and in the stroma, whereas only ERBB1 was detectable in the alveoli. With the onset of lactation, expression of ERBB1 was downregulated in all mammary cell types, while the expression of ERBB2 and ERBB3 was minimal. By contrast, others reported that ERBB2 expression was increased in the mammary glands during lactation, alongside the presence of an ERBB2-MUC4/sialomucin complex, although the localization of this complex depended on the antibody used [60, 61].

By contrast, there is limited data for the spatial distribution of ERBB ligands in the mammary glands of rats. In fact, a complete profile of gene expression only exists for *Tgfa* mRNA, which localized to both the ductal and alveolar epithelium, and to 10–25% of stromal fibroblasts, in the mammary glands of virgin female rats [62]. These findings contrast to those of others who found that *Tgfa* mRNA expression was abundant in myoepithelial cells and fibroblasts at various stages of mammary development, including in the glands of nulliparous rats [55]. Proteins for TGFα and EGF were also found in the epithelium, stroma and adipose tissue in the mammary glands of pubescent rats [63], while transcripts for *Areg*, *Ereg*, *Hbegf* and *Btc* were also detected in the mammary glands of adult virgin nulliparous rats [64]. Akin to what was seen in pubescent rats, *Tgfa* mRNA was present in all MEC of pregnant mice, and in only a small proportion of fibroblasts. With the onset of lactation, the expression of *Tgfa* was markedly increased, and was exclusive to the epithelium [62].

### Livestock Species

Several considerations emphasize how the normal mammary glands of livestock species might be more useful than those in rodents for studying the normal processes that dictate breast cancer risk. More specifically, domestic ruminants (cows, sheep and goats) are attractive models for study given the possibility of sampling repeated tissue and milk samples from single quarters of their udders. Furthermore, their mammary parenchyma is composed of TDLU that are also present in the human breast, but that are absent from the murine mammary gland. Likewise, different connective tissue components in the mammary glands of cattle and mice might explain why, when either human or bovine MEC were grafted into the mammary fat pads of mice, they failed to develop [65] versus mouse cells. Nevertheless, there is only sparse information regarding ERBB receptors in the mammary glands of ruminants (Tables 1, 2 and 3). From binding assays, ERBB1 was identified on purified plasma membranes from the mammary glands of pregnant and lactating cows [66]. Similarly, the capacity to bind TGFα was used to estimate the levels of ERBB1 in the mammary glands of sheep, which were higher in nulliparous females [67]. Lee and colleagues sought to describe the expression of ERBB receptors in the mammary glands of dairy cows prior to and during puberty (at 8 and 13 months of age, respectively). Expression of ERBB2, ERBB3, and to a lesser extent ERBB4, was detected in the mammary glands from both prepubertal and pubescent females, whereas the level of ERBB1 was much lower [68]. Despite several limitations of this study, the work highlights there may be a contrasting profile of expression for ERBB receptors in the bovine mammary glands compared to in mice.

Investigation of potential ERBB ligands in the mammary glands of ruminants has only focused on three members of the EGF-like protein family, namely EGF, TGFα and AREG. Evidence for the presence of EGF in the bovine mammary glands stems from a single study that reported elevated expression for both *EGF* and *TGFA* mRNA during mastitis [69]. In goats, EGF was also detected in prepartum mammary secretion [70], consistent with its presence in bovine colostrum [71]. Within the mammary glands of cows, *TGFA* was detected primarily at the onset of the dry period during regression and subsequent regeneration of the gland prior to parturition, while the presence of other transcripts suggested that other EGF-like factors may be expressed across all stages of mammary development [72, 73]. In contrast with those findings, Plath et al. (1997) described the highest levels of *TGFA* mRNA in the mammary glands of post-lactational non-pregnant cows during involution and, to a lesser extent, in the mammary glands of virgin heifers. In sheep, transcripts for *AREG* were detected in the mammary glands across a range of states, including in lambs, at late gestation (140d of a 150d gestation), and during lactation. The AREG protein localized to the nuclei and cytosol of luminal cells and in connective tissue of pregnant sheep, and in the nuclei of luminal cells in lactating sheep, but not in the mammary glands of female lambs [75].

The histomorphology of the mammary glands in female pigs displays striking similarities with that of the human breast [4], especially TDLU that are comparable, in both aspect and number, to those in humans. Even though a complete spatiotemporal description of ERBB receptors and ligands is not available for this species, our preliminary data indicate that the ERBB receptors and EGF-like ligands in the mammary glands of pigs are developmentally and hormonally coordinated.

### Carnivores

The scarce information about ERBB receptors and ligands in the normal mammary glands of canids is often contradictory, and mostly derives from studies investigating the role of ERBB receptors in mammary tumors. The ERBB1 protein was detected in both the normal [76–79] and cancerous mammary glands of bitches [78, 80–83], while gene expression for *ERBB1* has only been reported in mammary tumors [84]. Gama et al. localized membrane-associated and cytoplasmic staining for ERBB1 to myoepithelial cells and the perilobular stroma of the normal mammary glands. No variations in ERBB1 levels were apparent across physiological stages, except for a possible upregulation during estrus and the luteal phase [76]. Both ERBB2 protein and *ERBB2* mRNA were expressed in the normal mammary glands of bitches, predominantly in the epithelium [85, 86]. Whereas ERBB2 is commonly overexpressed in canine mammary tumors, there is no clear association between its protein status and histological grade [82, 87–89], clinical parameters [86, 88] or prognosis [90, 91]. Likewise, there are inconsistent relationships between the level of *ERBB2* mRNA and protein in both normal and cancerous tissues [85, 86, 92], perhaps reflecting either diversity among the studied populations or the use of different antibodies [87].

Mammary cancer in felids is commonly described as a model for HER2-overexpressing-hormone independent human breast cancers, which is why information about the ERBB family in these species almost exclusively focuses on ERBB2. Expression of ERBB2 in the normal mammary glands of queens, at both the mRNA and protein levels, ranges from absent to very low [93–96]. Higher epithelial immunoreactivity for ERBB2 was observed in malignant tumors compared to benign lesions, although not necessarily alongside higher gene expression [93, 95], or gene amplification [94, 97, 98].

### Humans

The ERBB receptors have long been implicated in the etiology of breast cancer. Overexpression of ERBB1, which is associated with increased tumor size and poor outcome, occurs in 15 to 30% of breast cancers [99]; ERBB1 is also overexpressed in anywhere from 13 to 76% of triple negative breast cancers [100–102]. Overexpression of ERBB2 occurs in 20–30% of breast cancers, often due to *ERBB2* gene amplification [103]. While mutations in *ERBB1* are rare, mutations in *ERBB2* do exist, leading to its constitutive activation that enhances cell growth and tumorigenicity. Overexpression of ERBB3 is also common in human breast cancers [104] and likely enhances ERBB1-signaling [105], whereas both the overexpression and downregulation of ERBB4 expression has been reported in mammary tumors. The ability of ERBB4 to both inhibit and promote epithelial proliferation in the normal mammary gland reflects its different roles in pathology, whereby ERBB4 expression is commonly related to a less aggressive phenotype, while activated ERBB4 can also promote proliferation, likely mediated by its intracellular domain [106].

Despite a clear role for ERBB receptors and their overexpression in breast cancers, much less is known about their biology in the normal human breast (Tables 1, 2 and 3). Certainly, ERBB1 is expressed in the normal breast given its transcript was detected in epithelial cells isolated from nonpregnant, nonlactating women [107, 108]. In normal tissue and hyperplastic or cancerous mammary lesions, ERBB1 localized to myoepithelial cells, alongside its variable presence in the normal epithelium [109–111]. Consistent with the presence of ERBB1, the ERBB1-ligands AREG and TGFα are either transcribed or translated by cultured normal breast epithelial cells [107, 108, 112], and *TGFA* levels were increased in pregnant as compared to nulliparous woman [62]. Moreover, *EREG* gene expression was specifically induced in the mammary glands of premenopausal women during the luteal phase, suggesting that EREG may mediate the actions of P in the human breast [113]. Regarding the role of ERBB1 in normal mammary glands, the activation of ERBB1 was required for differentiation of the myoepithelial lineage in immortalized human progenitor MEC that could differentiate into both luminal and myoepithelial cells in the presence of EGF. By contrast, AREG could only induce the luminal lineage, while TGFα only induced the myoepithelial lineage [114, 115]. Despite an absence of data regarding the role of ERBB-mediated signaling during lactogenesis, both EGF and HBEGF were detected in human milk and were associated with a protective effect against intestinal injury, which may further imply a potential role for all ERBB homo- and heterodimers in neonatal gut health [116].

## Function of ERBB and Their Ligands in the Developing Mammary Glands: A Family Drama

### Puberty and Maturity

#### Rodents

The mammary glands of pubescent mice and rats initiate their allometric development around 4 and 7 weeks of age respectively, as the ductal epithelium ramifies into the surrounding stroma. In mice there is a clear role for both E and P in developing the ductal network, whereas the mammary glands of virgin rats develop a more ductal-lobular morphology that is insensitive to P [117, 118]. Several lines of evidence indicate that this hormone-directed ductal expansion is mediated by local ERBB receptors and their ligands that not only amplify and modulate upstream systemic stimuli, but also confer variable morphogenic responses (Fig. 1).

Aspects of this ductal morphogenesis have been explored in vitro with a focus on ERBB1-dependent signaling. Different studies using primary cells and organoids from virgin mice highlighted a role for both EGF and TGFα in promoting ductal development [119, 120], and the potential for EGF to synergize with either P or PRL [121]. Another aspect highlighted by these experiments in vitro is that the regulation of ERBB-signaling likely depends on the composition of the mammary stroma. In fact, EGF-stimulated MEC produced their own basement membrane (BM) when cultured on collagen I-coated dishes, while deposition of BM was impaired on collagen IV, alongside the downregulation of ERBB1 [122].

A role for ERBB1 as a downstream effector of systemic hormone action in the mouse mammary gland in vivo is undisputed. Mice overexpressing a C-terminal-truncated ERBB1 had restricted growth of their mammary ducts as early as 5 weeks of age that was accompanied by fewer and smaller TEBs, followed by decreased ductal branching and density by 16 weeks [123]. On the other hand, mammary stroma expressing wild type ERBB1 could sustain the branching of either wildtype or *Erbb1*-null epithelium [43, 46, 124], consistent with the localization and activation of ERBB1 in the periductal stroma [9, 45]. There is also likely a role for multiple EGF-like ligands during this stage of development. Although EGF, TGFα and AREG all promote ductal morphogenesis, only AREG is indispensable for ductal growth [48]. This finding aligns with the demonstration that *Areg* is the only ERBB-ligand that is upregulated by E during puberty, and that shedding of AREG by ERα-positive epithelial cells, as induced by ADAM17, is essential for activating ERBB1 in the stroma prior to the stimulation of ductal elongation [1, 46]. However, questions remain as to how these receptors and ligands mediate the local response to various endocrine cues. First, additional endocrine or local factors besides E likely regulate the local synthesis of AREG. For example, both E and P can induce ductal growth and TEB formation in ovariectomized peripubertal mice, where P-induced development is associated with increased AREG expression, and is blocked by the inhibition of ERBB1 [125]; moreover, the recruitment of macrophages and eosinophils into the developing mammary glands of pubescent mice is triggered by both E and P, and is likely mediated by AREG-induced nuclear factor kappa B (NF-kB) - dependent cytokine expression [126]. Second, ERBB1 is also present in the epithelium, raising questions about its specific function in that cell type. Furthermore, the presence of other ERBBs in the epithelium may create potential heterodimerization partners for ERBB1, consistent with the demonstration that ductal development is impaired when epithelial *Erbb1* is silenced. Indeed, the transplantation of *Erbb2*-null mammary epithelium into the cleared mammary fat pad of wildtype mice led to impaired ductal development, delayed TEB appearance (despite an increased number of branch points) and severe disruption of TEB histomorphology that corresponded to altered distribution of smooth muscle actin, as well as P- and E-cadherins [127]. A similar phenotype was recorded when epithelial expression of *Erbb2* was silenced [128]. However, as mentioned earlier, ERBB2 is an orphan receptor, raising the prospect that either heterodimerization of epithelial ERBB1 with ERBB2 plays a minor role in mammary development during puberty, as proposed by Sternlicht et al. [46], or that ERBB3 and/or ERBB4 fulfil specific roles during this stage. Indeed, when *Erbb3*-null mammary epithelium was transplanted into the wildtype mammary fat pad, the developmental deficits were comparable to those seen in *Erbb2*-null transplants, and persisted into adulthood [129]. Reduced outgrowth of the ductal network was also recorded in mice homozygous for *Erbb3*^Δ85^ (an allele lacking PI3K-binding sites), further confirming a role for the ERBB3/PI3K pathway during glandular morphogenesis [130]. Given these findings, the hypothesis that ERBB3 functions as an ERBB2 heterodimerization partner is further supported by the demonstration that implants of heregulin (isoform of NRG1) could induce ductal development in prepubescent mice [131], and could stimulate ductal morphogenesis even when *Erbb1* was deleted [46]. Among the various ERBB, there are also reasons to speculate that ERBB4 may serve a more precocious role than generally acknowledged. Indeed, the Cyt-1 and Cyt-2 splice variants of ERBB4 can either inhibit growth while enhancing differentiation, or promote proliferation. For example, overexpression of ERBB4-Cyt-1 (either as its full-length form, or as the intracellular domain of JMa-Cyt-1) accounts for the anti-proliferative effects of ERBB4 during puberty that results in suppressed ductal elongation and TEB development, followed by enhanced lobuloalveolar budding in sexually-mature female mice. The overexpression of Cyt-2 (intracellular domain of ERBB4) caused hyperproliferation of epithelial cells in both ducts and lobuloalveoli, which did not occur with overexpression of full length JMa-Cyt-2, suggesting that the membrane-associated ERBB4 might interact with the Cyt-2 intracellular domain [132, 133]. Taken together, these findings suggest that, despite the low level of ERBB4 expression in the mammary glands during puberty, ERBB4 might serve a mild growth-suppressing effect on the epithelium during this phase, followed by a potential differentiative effect during gestation and lactation. Interestingly, Schroeder et al. only detected ERBB3 protein in the mammary glands of pregnant mice, while the expression of ERBB4 in the mammary glands during puberty was uncertain [9]. Combined, these observations highlight the need for a more detailed spatiotemporal analysis of ERBB receptors in the murine mammary gland.

Lastly, there is a compelling likelihood that other EGF-like factors besides AREG also contribute to mammary gland development during puberty. For instance, EGF and TGFα both induce ductal morphogenesis in vivo [45, 54, 134]. Furthermore, both inflammatory and ERBB1-signaling related molecules such as *Erbb1*, *Hbegf* and *Adam17* were upregulated in a unique E-independent phenotype of ductal growth induced by dietary conjugated linoleic acid [135]. Indeed, E-independent activation of ERBB1 by AREG or other growth factors such as HBEGF might contribute to normal mammary gland development, an effect that may be even more pronounced during obesity-associated inflammation that promotes tumorigenesis of certain E-independent cancer subtypes.

As previously mentioned, all ERBB receptors were detected in the mammary glands of pubertal and adult virgin rats, consistent with the effect of ERBB-dependent signaling on both growth and differentiation of mammary epithelial organoids from virgin rats cultured in reconstituted BM [58, 59]. Similar to findings in mice [125], the expression of *Areg* in the mammary epithelium of 18 week old virgin rats could be stimulated by either E or P, but was maximal in response to their combination. Crosstalk between E, P and ERBB1 was also proposed whereby AREG (released in response to E and P) and P (leading to activation of the P receptor-B) could synergize through various ERBB1-downstream signaling pathways to stimulate epithelial proliferation [64]. Interestingly, in the same study gene expression for other ERBB1 ligands was modulated by either E or P. Specifically, *Ereg* and *Btc* were upregulated by E, whereas *Tgfa* was induced by P, raising the possibility that TGFα may facilitate cross talk between activated P receptor-B and signaling downstream of ERBB1.

#### Ruminants

Allometric growth of the mammary epithelium in nulliparous female cows (heifers) commences at around 2–3 months of age and prior to puberty, or potentially earlier [136, 137]. Similarly, allometric growth in female sheep can start as early as 4 weeks of age [138]. By the time of puberty (approximately 8 months of age for sheep, and 11 months for cattle), the epithelial ducts have elongated into the surrounding mammary fat pad, which is more fibrous than the predominantly adipose stroma of mice. The mammary parenchyma in pubertal ruminants is characterized by a complex ductal-lobular parenchyma surrounded by distinguishable inter- and intralobular connective tissues [139] (Fig. 1). These unique histomorphological and tissue interactions raise the prospect for differential roles for ERBB receptors and their ligands in these species, like may also occur in humans, and distinct from those in model species such as mice.

Evidence in vitro points to roles for EGF, TGFα and AREG in the regulation of mammary growth in ruminants. When comparing ligands, recombinant human EGF (rhEGF) was more effective than rhTGFα for stimulating the proliferation of bovine MEC [140], whereas both were more effective than rhAREG. By contrast, rhTGFα promoted DNA synthesis in alveolar epithelial cells from nulliparous ewes in a dose-dependent manner more efficiently than rhEGF, independent of developmental stage, although DNA synthesis was delayed in cells from nulliparous females versus those that were pregnant [67, 141]. Similarly, rbTGFα stimulated greater proliferation by bovine MEC than did rhEGF [73, 142, 143], raising the question of whether this different outcome reflected that the cells were from a different physiological state, or whether there is species-specificity at play for human and bovine TGFα. Beyond its direct actions, EGF synergized with insulin-like growth factors (IGF-I, IGF-II and des3-IGF-I) to induce mitosis in undifferentiated MEC in collagen [144, 145], while TGFα synergized with insulin (INS) to induce DNA synthesis in MEC from nulliparous ewes [141].

The positive effect of EGF on ruminant mammary epithelium was confirmed by transplantation of mammary tissue from mid-pregnant heifers into ovariectomized-sialoadenectomized athymic mice. In this model, EGF had almost no effect on epithelial proliferation, whereas it synergized with the combination of E and P to restore the level of DNA synthesis back to that in ovary-intact, steroid-treated mice [146]. These findings raise the prospect that additional interactions between ligand-stimulated proliferation and ovarian hormones, as well as perhaps the nature of the stroma, create a stage- and tissue-specific growth-modulating environment for the mammary epithelium.

### Gestation

The mammary glands undergo pronounced and rapid changes during gestation - not only through extensive proliferation of the epithelium that gives rise to alveoli arising from the ductal structures, but also cytologically and biochemically in preparation for the onset of lactation. In this section we review the potential roles for ERBB receptors and their ligands in the mammary glands across various species, while encouraging the reader to keep in mind that the final transition during secretory activation occurs alongside terminal differentiation of the mammary epithelium and a final wave of proliferation.

#### Rodents

There is clear in vitro evidence that signaling downstream of the ERBB receptors can direct lobuloalveolar development and differentiation of the mammary epithelium in rodents. One illustration of this effect is in whole organ culture, where a first wave of lobuloalveolar development can be induced in mammary glands from steroid-primed pubescent mice following their exposure to INS, PRL, aldosterone and hydrocortisone (HC), while EGF is essential for triggering a second wave of growth [147]. In testing the interrelationships between these hormonal regulators, others investigated the interactive responses of the mammary epithelium to EGF and PRL. Interestingly, PRL inhibited EGF-dependent DNA synthesis in various models in vitro, while also promoting the transcription of *Egf* itself [148, 149]. Surprisingly, EGF and PRL both independently upregulated the pre- and post-transcriptional levels of β-casein, whereas their combination, in the presence of INS and HC, had the opposite effect [55, 149, 150]. Some have proposed that this negative interaction might be an artefact of a supraphysiological concentration of INS [151], which aligns with evidence showing that EGF enhances the secretion of β-casein in the presence of PRL and dexamethasone [152]. A combined interpretation of these studies is that EGF coordinates epithelial proliferation and differentiation, while PRL progressively inhibits this effect and simultaneously promotes the transcription of *Egf* as an autocrine factor to sustain secretory differentiation. Variation in the relative abundance of ERBB1 homo- and heterodimers in MEC during this period may well also account for the switching of EGF towards a pro-differentiative function. Neuregulins also specify the functional state of MEC in vitro, as was revealed using antisense nucleotides against endogenous *Nrg*; these inhibited lobuloalveolar development in cultured whole mammary glands, whereas exogenous *Nrg* reversed this inhibitory effect, leading to increased expression of β-casein and WAP [44].

**Fig. 3:**
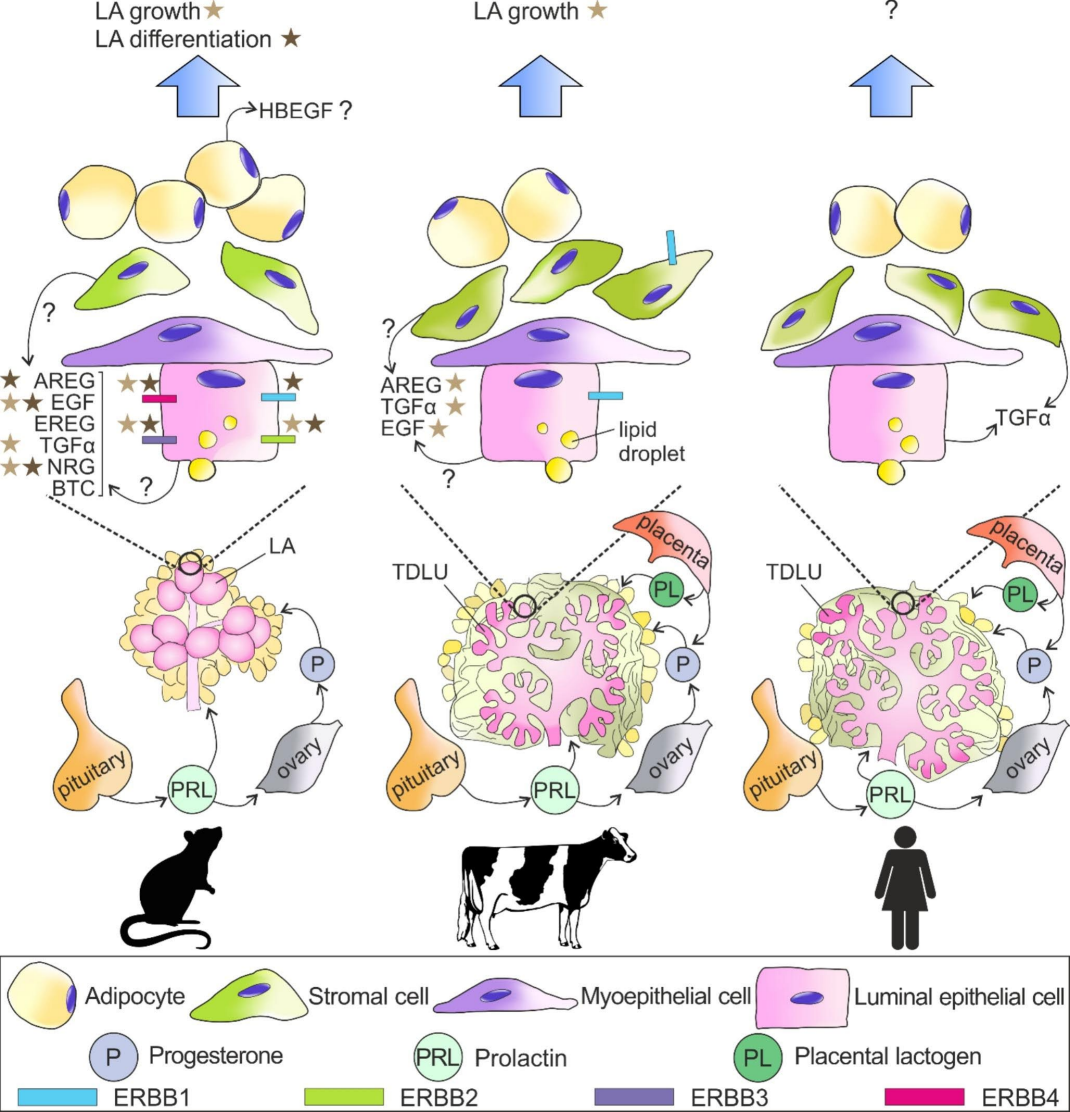
Mammary gland histomorphology and the epidermal growth factor (EGF) receptor (ERBB) family of receptors and their ligands in mice (left), ruminants (middle) and humans (right) during pregnancy and lactation. The main systemic hormones involved during gestation and lactation are summarized. Asterisks link individual ERBB receptors to specific biological effects (blue arrows). Question marks represent incomplete information regarding ERBBs and their ligands. Abbreviations = AREG, amphiregulin; BTC, betacellulin; EGF, epidermal growth factor; EREG, epiregulin; HBEGF, heparin-binding EGF; NRG, neuregulin; Pituitary, pituitary gland; TDLU, terminal ductal lobular unit; TGFα, transforming growth factor-α. Abbreviations for graphics are explained in the figure. Figure created with CorelDraw X8.

Evidence in vivo also suggests that ERBB1 and ERBB2 might support lobuloalveolar development during gestation, despite being unable to compensate for the effects of *Erbb3* deletion [49] (Fig. 3). As a matter of fact, mice overexpressing a dominant negative-*Erbb2* in their mammary glands displayed abnormal, condensed lobuloalveoli by the end of gestation [51]. Furthermore, several ERBB1-ligands are essential during this developmental stage when deletion of *Areg* leads to impaired lobuloalveolar development, which is exacerbated by the additional deletion of *Egf* and *Tgfa* [40]. More specifically, TGFα was more effective than EGF for inducing lobuloalveolar growth [146, 147]. Unlike ERBB1 and ERBB2, an indispensable role exists for ERBB3 during gestation, as evidenced by the fact that *Erbb3*-null mice have defective lobuloalveolar expansion, decreased epithelial proliferation and enhanced apoptosis of luminal epithelial cells, together with impaired activation of AKT and STAT5. Intriguingly, failed proliferation and differentiation of the mammary epithelium upon the loss of ERBB3 recovered during late gestation and early lactation, while phosphorylation of ERBB4 (Tyr1056) increased [49, 129]. In fact, ERBB4 might compensate for the defects induced by the loss of ERBB3 in late gestation, where the ERBB4 isoform JMa-Cyt-1 (the full-length, antiproliferative form of ERBB4), which harbors a PI3K-binding site at Tyr1056, would likely heterodimerize with ERBB3 and induce differentiation.

Not by chance, ERBB4 plays a major role in the mammary glands during gestation, moreso than other ERBB receptors. Lactating primiparous *Erbb4*-null mice have scarce lobuloalveolar differentiation with reduced activation of STAT5 shortly after parturition, likely due to deficiencies that arose during gestation [153]. Multiparous female mice harboring Cre-lox mediated deletion of both epithelial *Erbb4* alleles had severely-compromised lobuloalveolar growth as early as day 13.5 of gestation, due to both impaired proliferation and defective differentiation [50]. This dual proliferative and differentiative effect for ERBB4 might reflect the opposing functions of ERBB4-Cyt-1 and Cyt-2 isoforms (derived by the alternative splicing of the ERBB4 intracellular domain), as evidenced in HC11 cells overexpressing Cyt-1 or Cyt-2 ICD isoforms that undergo apoptosis or proliferation, respectively [133]. The sparse ductal development described in pubertal mice overexpressing ERBB4-Cyt-1 (either the intracellular domain Cyt-1, or the full-length isoform JM-a-Cyt-1) extended into mid-gestation, after which developmental deficiencies abated. On the other hand, epithelial cells in ducts and lobuloalveoli did not proliferate when the full-length isoform JM-a-Cyt-2 was overexpressed in the mammary glands, perhaps because the membrane-bound fraction of ERBB4-Cyt-2 had a compensatory effect. An interesting model was proposed whereby epithelial expression of Complement subcomponents Clr/Cls, Uegf, Bmpl and zona pellucida-like domain-containing protein 1 (CUZD1) would complex with STAT5 following activation of the PRL receptor (PRLR), increasing local expression of NRG1, EREG (both increased in the mammary gland during gestation [9]) and EPGN; in turn, these ligands would promote epithelial proliferation and differentiation via ERBB1 and ERBB4 [154]. Such an interaction between ERBB4, PRLR and STAT5 would not only enhance the compensatory role of ERBB4 in lobuloalveolar expansion and differentiation (as hypothesized by Williams et al. [49]), but might also partially account for the unique differentiative properties of ERBB4, which may override ERBB1-dependent growth [155]. Finally, there is a clear positive role for NRG1 during lobuloalveolar development, consistent with the pro-differentiative effect of ERBB3 and ERBB4. Specifically, deficiency of the NRG1 isoform NRG1-α leads to the abnormal condensation of lobuloalveoli and decreased epithelial proliferation [156].

#### Ruminants

Pregnancy in heifers and ewe lambs is typically initiated at 15 months and 12–18 months of age, with corresponding gestation lengths of about 9 months and 5 months, respectively. Extensive alveologenesis and stromal remodeling in the mammary glands of ruminants, including in cattle [157] and small ruminants [158, 159], occurs primarily during gestation, while mammary DNA content remains constant after parturition. There are several lines of in vitro evidence to support a role for the various EGF-like ligands in the mammary glands of pregnant ruminants. Both TGFα and EGF are mitogenic for cultured MEC from pregnant cows [73]. Likewise, treating explants from midpregnant Holstein heifers with EGF or TGFα was sufficient to induce maximal DNA synthesis, despite suboptimal activation of ERBB1 [160]. In a similar way, MEC from pregnant ewes initiated DNA synthesis in response to EGF, TGFα, or AREG; what is more, TGFα compensated for the inhibition of DNA-synthesis induced by heparin. On the other hand, heparin maximized the synergistic response to TGFα and either INS or IGF-1, probably by decreasing the interactions between AREG and INS/IGF-1. In essence, a complex yet unresolved interplay between EGF-like ligands and IGF-related ligands seems to occur during gestation, with the potential for modulation by heparin-like molecules [141].

Other approaches have also been used to demonstrate the mitogenic effects of various EGF-like ligands in the mammary glands of ruminants during gestation in vivo (Fig. 3). Both hEGF and bTGFα induced mammary growth and DNA synthesis in late pregnant cows when they were instilled into half the udder quarters [143]. In a different study, explants of mammary tissue from mid-pregnant cows were transplanted to mice, which were then primed with steroid hormones. In this model, PRL and HC, alone or synergistically, decreased EGF-dependent DNA-synthesis by affecting both abundance and kinase activity of ERBB1. An intriguing question that warrants investigation is whether PRL can simultaneously synergize with EGF to induce epithelial differentiation in the mammary glands of ruminants, as proposed in rodents [160–162].

### Lactation

#### Rodents

As outlined above, ERBB4 is indispensable for terminal differentiation of the mammary gland, as highlighted by the fact that *Erbb4*-dominant negative mice had distended ducts bearing persistently condensed, lipid-filled lobuloalveoli, as well as reduced epithelial proliferation and expression of β-casein, WAP and α-lactalbumin [50, 52, 153]. These defects coincided with impaired activation of STAT5, potentially due to crosstalk between the PRLR and ERBB4 [155]. Not surprisingly, pups nursed by either *Erbb4*-null dams or those expressing a dominant-negative *Erbb4* in their mammary glands had higher mortality and lower body weight due to inadequate milk production by the dams [50, 153]. While the activation of ERBB4 in the mammary glands is crucial for lactation, its requirement is not exclusive (Fig. 3). Specifically, the phenotype of immature, condensed lobuloalveoli filled with fat droplets and scarce proteinaceous material also existed in transgenic *Erbb2*-dominant negative mice, supporting the notion that ERBB2 might normally heterodimerize with ERBB4 to facilitate terminal differentiation of the alveolar epithelium [51]. Moreover, defects in the mammary glands of *Erbb3*-null mice appeared primarily around the end of gestation, then progressively improved around parturition, perhaps due to the concurrent upregulation of ERBB4 [49]. However, this proposal requires further validation, given that mice homozygous for *Erbb3*^Δ85^ have defective proliferation and differentiation within their mammary glands (giving rise to thicker epithelium, less expanded alveoli, decreased β-casein and WAP protein expression and secretion) that persists to day 3 of lactation [130]. Lastly, the presence of mutated *Erbb1*, as found in waved-2 mice, is associated with smaller mammary glands, a lower parenchyma/fat ratio, decreased secretions and vacuolation, and higher mortality of pups [163]. A role for ERBB1 also extends to the health of the neonate, where *Erbb1*-null newborn mice developed hemorrhagic enteritis [164], while EGF supplementation reduced the incidence of necrotizing enterocolitis in newborn rats [165].

Given the potential role(s) for all ERBB receptors during lactation, it is perhaps not surprising that several EGF-like ligands have also been implicated during this period. For example, a deficiency of NRG1 led to the distention of scarcely arranged lobuloalveoli, alongside decreased expression of β-casein and less epithelial proliferation [156], similar to the phenotype in *Erbb4*-dominant negative mice. Meanwhile, the correlation between TGFα expression and lactation efficiency remains controversial. Specifically, whereas TGFα enhanced progression towards terminal differentiation when it was overexpressed in the mammary glands, in association with precocious lobuloalveolar development (reviewed by Schroeder and Lee [166]), lactation was often impaired in those animals, and multiparous mice often developed hyperplastic lesions. What is more, the extensive lobuloalveolar development in mice overexpressing TGFα was not associated with the expression of milk proteins such as WAP [53, 167], nor with the accumulation of secretory products [131]. One possible explanation for how transgenic overexpression of TGFα suppresses lactation potential is that TGFα impairs the upregulation of PRLR expression around parturition [168]. In the same vein, simultaneous overexpression of TGFβ (a mammary growth suppressor) and TGFα led to the TGFα-transgenic mice having normal mammary glands, but impaired lactation [169]. It should be noted that the role for steroid hormones was not isolated in all the studies, making it difficult to define the local, TGFα-dependent effects on mammary differentiation [170]. In summary, TGFα likely supports terminal differentiation in gestation, but a successful lactation reflects a precise balance between progressive downregulation of TGFα, PRLR and steroid receptor interactions, and the local expression of other growth factors.

#### Ruminants

There is a paucity of data regarding whether signals downstream of epithelial ERBB receptors affect lactational competence in ruminants (Fig. 3). Some evidence indicates that EGF accumulates in the mammary glands of pre-parturient cows [71] and small ruminants [70, 171], while EGF is detectable in milk at the beginning of lactation, but then declines afterwards. While there was a 10-fold increase in the amount of EGF in the colostrum of lactating ewes a day after parturition, intravenous injection of EGF had no effect on the body weight of their lambs, on colostrum composition, or on milk yield [171]. As in other physiological stages, TGFα is more potent than EGF for inducing DNA synthesis in MEC from lactating ewes, especially in synergy with INS or IGF-1 [141].

### Involution

Upon weaning there are massive changes within the gland, including milk stasis and degradation of its components, epithelial apoptosis, remodeling of the extracellular matrix, and phagocytosis, that all proceed over time to varying extents across species. While the first, reversible phase of involution is acutely responsive to milk statis, leading to the up- or downregulation of epithelial STAT3 and STAT5 respectively, the second phase is irreversible and reflects the involvement of systemic factors and macrophages. We posit that activated ERBB receptors are involved in both phases, particularly by virtue of their ability to trigger STAT signaling and to mediate macrophage-dependent tumor cell motility [172].

#### Rodents

Activated ERBB3 and ERBB4 apparently counteract remodeling of the mammary gland, as demonstrated by accelerated involution in transgenic mice homozygous for *Erbb3*^Δ85^ (lacking PI3K-binding sites) [130] and those harboring the epithelial deletion of *Erbb4* [50]; in the latter case the remodeling defects are reminiscent of altered AKT signaling. Conversely, deletion of *Erbb2* in the mammary epithelium was not constantly associated with defective involution [128].

It is perhaps not surprising that overexpression of TGFα in the mammary glands of mice impairs epithelial apoptosis after parturition (as opposed to the pro-apoptotic effect of TGFβ1) given its aforementioned mitogenic effect during earlier developmental stages [173]. A significant decrease in the abundance of *Nrg1* and *Nrg2* transcripts was recorded in the involuting mammary glands of heterozygous p63 transgenic mice, consistent with the pro-survival activity of ERBB3 and ERBB4. As a matter of fact, p63 exerts a pro-survival effect on basal cells during early involution by counteracting both the STAT3 and p53 pathways, while promoting the transcription of *Nrg1* and *Nrg2*, which are activators of the ERBB4-STAT5 pathway [174].

#### Ruminants

Lobuloalveolar regression during involution is never complete in ruminants [175], particularly in dairy cows where lactation and gestation overlap. Surprisingly, there are few insights as to the role of ERBB receptors and their ligands during involution of the mammary glands in ruminants. The levels of *TGFA* were high in the involuting mammary glands of dairy cows [72], which could potentially contribute to the sustained lobuloalveolar phenotype. In vitro, EGF had an antiapoptotic role on BME-UV cells cultured in FBS-deficient medium, which might reflect a compensatory pro-survival effect in the mammary gland during involution [176]. Interestingly, the transcription of both *EGF* and *TGFA* was increased during mastitis [69]. Given the economic impact of this condition on the dairy industry, the role for ERBB-signaling during mastitis and the associated tissue inflammation and repair warrants further investigation.

### ERBBs in Dogs and Cats: Only Background Actors?

The mammary glands of dogs and cats are often proposed as models for human breast cancer. Indeed, specific cancer subtypes such as the spontaneous feline carcinoma, which is a possible model of HER2 overexpressing breast cancer [93], and canine hormone-dependent cancers [177], share many similarities with the human disease. These analogies, together with a high incidence of ERBB1 and HER2 overexpression in both canine and feline mammary cancers, explain the considerable interest in ERBB signaling in veterinary medicine. Ironically, almost nothing can be said about the contribution of ERBB receptors towards the physiological development of the canine or feline mammary glands. Logic would suggest that the ERBB receptors have a dynamic profile of expression across the course of mammary development in carnivores, as in mice. Since a major unsolved question in veterinary oncology is whether a precocious ovariectomy significantly decreases the incidence of mammary cancers [178], the contribution of ERBB-signaling around the time of spaying might be a main factor affecting tumorigenesis in our pets.

## Behind the Scenes: ERBB-signaling

The many combinations of ERBB receptors and their ligands, ERBB positive/negative effectors, and potential biological outcomes, highlights how ERBB-signaling is a multilayered signaling network [15]. Defining these pathways is critical for understanding the main interactions and their outcomes. To complete the script, we will briefly review the principal signaling pathways that can be activated by homo- or heterodimerizing ERBB receptors, and their contribution during normal mammary development and morphogenesis.

### RAS/ERK Pathway

ERBB1, -B2 and -B4 all activate the RAS/ERK pathway due to their multiple binding sites for Grb2 and SHC. The final effector of this signaling cascade, ERK, can either translocate to the nucleus, or activate additional transcriptional factors, apoptosis regulators or hormone receptors. Activation of the RAS/ERK pathway yields a range of phenotypes including proliferation, differentiation, and survival, as well as angiogenesis and cell motility, which all occur in the mammary gland [13, 179]. Unique combinations of ERBB and RAS/ERK may function during mammary gland development. For example, the formation of mammospheres by duct/limited progenitor cells in the murine mammary gland was mediated by ERK1/2, presumably through the release of AREG [180]. In addition, the formation of ductal branches arising from cultured mouse and rat primary MEC in response to TGFα, EGF or AREG was also ERK-dependent [64, 120, 181]. Moreover, specification of the myoepithelial lineage in human breast organoids, which is triggered by EGF-activated ERBB1, depends on ERK1/2 [115]. Based on these findings, activation of the RAS/ERK pathway downstream of ERBB receptors should be essential for mammary development from puberty until adulthood. Indeed, Schroeder and colleagues [9] showed that activated ERK1/2 colocalized with ERBB1 and ERBB2 in the mammary glands of virgin mice, while it was almost absent during gestation and lactation. On the other hand, transgenic mice overexpressing TGFα and/or PRL revealed that these peptides cooperate to activate ERK1/2 resulting in a number of developmental abnormalities, while also rendering the mammary epithelium insensitive to E [182]. The significance of this relationship in the mammary glands of pregnant and lactating animals remains to be addressed. This synergy between TGFα and PRL might involve the modulation of ERBB3 and/or ERBB4 heterodimers, given that the levels of ERBB1 (the only TGFα receptor) are the same in TGFα and TGFα/PRL bitransgenic mice [183].

### PI3K/AKT Pathway

Both ERBB3 and the JMa-Cyt-1 isoform of ERBB4 can activate PI3K/AKT signaling due to their multiple binding sites for the p85 subunit of PI3K. Alternatively, PI3K can also be indirectly activated by RAS. Meanwhile AKT, which plays a central role in signaling downstream of ERBB receptors, affects a number of biological processes crucial for mammary gland development, including glucose metabolism, cell survival, protein synthesis, and cell motility [13, 184]. As previously mentioned, activated ERBB3-ERBB4 heterodimers and AKT can interact to direct mammary growth and differentiation. Indeed, the outgrowth of mammary ducts was severely impaired in mice bearing alleles for ERBB3 that lacked P85a subunits [130]. AKT is also an essential mediator for lobuloalveolar expansion during late gestation, as evidenced by the fact that scant lobuloalveolar development in pregnant *Erbb3*-null mice was also associated with impaired activation of AKT. Furthermore, the ERBB4 isoform JMa-Cyt could partially reverse the lobuloalveolar defects and induction of AKT. While there are no data regarding the role of ERBB-AKT interactions during mammary development beyond gestation, these are likely to be decisive given that the activation of AKT1 and AKT2 isoforms can lead to either delayed or accelerated involution [185, 192, 193].

### STATs

The ERBB receptors can activate STAT proteins both directly and indirectly through JAK, which in turn phosphorylates STATs prior to nuclear translocation. Different STAT proteins have precise expression patterns and occupy distinct roles during mammary development [186], although evidence for an interaction between STAT and ERBB proteins only exists for late gestation and lactation. During these stages, STAT5 serves as a hub to signal lobuloalveolar development [187] and milk protein gene expression [188]. Both *Erbb3*-null [49] and *Erbb4*-null [50] mice have downregulated STAT5 in their mammary glands, together with the aforementioned developmental and lactogenic defects. While ERBB3 seems unable to induce phosphorylation of STAT5, there is the potential for ERBB4 to be a strong activator. In fact, not only is ERBB4 activated downstream of pSTAT5 in the mammary glands of mice [154], but it can also synergize with the PRLR to activate STAT5 in HC11 cells [131], where the interaction of ERBB4 and STAT5 in the epithelium is crucial for lobuloalveolar development. On the other hand, the ERBB1-dependent phosphorylation of STAT5 is indispensable [189] only in the stroma of pregnant and lactating mice.

During involution, STAT3 is constitutively expressed, but is specifically phosphorylated for its role in apoptosis and remodeling after weaning [190]. While there is no clear link between elements of ERBB-signaling and STAT3 during involution, there is evidence from multiple breast cancer lines that ERBB2-ERBB3 heterodimers can activate STAT3 upon NRG binding, in a signaling network that also involves the PR and the ERK1/2 pathway [191]. In a similar way, STAT1 expression peaks during involution, although its role is not entirely clear [192], while STAT5 is downregulated. Despite being expressed in the mammary glands throughout development, expression of STAT6 is upregulated during gestation, when it contributes to expansion of the alveolar lineage [193]. There is no information about how STAT6 and ERBB receptors interact during these stages, although transactivation of the EGFR by STAT6-mediated release of HBEGF has been described in peritoneal macrophages of mice [194].

## Conclusion

The family of characters reviewed here - the ERBB receptors and their ligands - are as complex and dynamic as any human family! A wide body of evidence highlights how in all species the ERBB family finds its strength through the interplay between different ERBBs and their ligands, rather than through the exclusive role for any one receptor or ligand at any stage. On the other hand the literature, albeit patchy, highlights that the biological functions of these molecules differs across species; a case in point being that there is a more diverse expression pattern for the ERBBs and their ligands in the mammary glands of rats and ruminants relative to in mice. In the same way, the relative importance of various EGF-like ligands in regulating mammary development appears to differ across species. We propose that the distinctive stromal composition of the mammary glands is an underlying regulator of ERBB biology and function in this context (Figs. 1 and 3). Finally, we suggest that mediation of upstream hormonal cues by ERBB receptors helps confer the range of histomorphological phenotypes across species. Central to this hypothesis could be the actions of P, which independently is non-proliferative in the mammary glands of primates, ruminants, rats and pigs [4, 195, 196]. While there is mixed information about whether P alone is proliferative in humans [197], the breast is also characterized by a complex ductal-lobular morphology. Elucidating how ERBB signaling converges with the actions of systemic hormones, both on the epithelium and the surrounding stroma, could further define the regulation of breast morphogenesis. At the same time, many unanswered questions remain regarding the role of ERBB receptors and ligands in veterinary pathology, where a deep understanding of their biology would help to identify better targets for treatments against mammary cancers in dogs and cats, as well as against mastitis in dairy animals and even humans.

All in all, while the family of ERBB receptors and ligands is now aged and mature, its members remain partly confused on the mammary gland stage. Like in Pirandello’s theatrical piece *Six Characters in Search of an Author*, mammary biologists are now asked to give each of these *fifteen* characters a voice and to fully unravel the roles they are playing in the mammary gland.
